# Measuring Cation Dependent DNA Polymerase Fidelity Landscapes by Deep Sequencing

**DOI:** 10.1371/journal.pone.0043876

**Published:** 2012-08-22

**Authors:** Bradley Michael Zamft, Adam H. Marblestone, Konrad Kording, Daniel Schmidt, Daniel Martin-Alarcon, Keith Tyo, Edward S. Boyden, George Church

**Affiliations:** 1 Department of Genetics, Harvard Medical School, Boston, Massachusetts, United States of America; 2 Biophysics Program, Harvard University, Boston, Massachusetts, United States of America; 3 Northwestern University, Departments of Physical Medicine and Rehabilitation, Physiology, and Applied Mathematics, Chicago, Illinois, United States of America; 4 Media Lab, Massachusetts Institute of Technology, Cambridge, Massachusetts, United States of America; 5 Department of Biological Engineering, Massachusetts Institute of Technology, Cambridge, Massachusetts, United States of America; 6 Department of Chemical and Biological Engineering, Northwestern University, Evanston, Illinois, United States of America; 7 McGovern Institute, Massachusetts Institute of Technology, Cambridge, Massachusetts, United States of America; 8 The Rehabilitation Institute of Chicago, Chicago, Illinois, United States of America; 9 Wyss Institute, Harvard University, Boston, Massachusetts, United States of America; Center for Genomic Regulation, Spain

## Abstract

High-throughput recording of signals embedded within inaccessible micro-environments is a technological challenge. The ideal recording device would be a nanoscale machine capable of quantitatively transducing a wide range of variables into a molecular recording medium suitable for long-term storage and facile readout in the form of digital data. We have recently proposed such a device, in which cation concentrations modulate the misincorporation rate of a DNA polymerase (DNAP) on a known template, allowing DNA sequences to encode information about the local cation concentration. In this work we quantify the cation sensitivity of DNAP misincorporation rates, making possible the indirect readout of cation concentration by DNA sequencing. Using multiplexed deep sequencing, we quantify the misincorporation properties of two DNA polymerases – Dpo4 and Klenow exo^−^ – obtaining the probability and base selectivity of misincorporation at all positions within the template. We find that Dpo4 acts as a DNA recording device for Mn^2+^ with a misincorporation rate gain of ∼2%/mM. This modulation of misincorporation rate is selective to the template base: the probability of misincorporation on template T by Dpo4 increases >50-fold over the range tested, while the other template bases are affected less strongly. Furthermore, cation concentrations act as scaling factors for misincorporation: on a given template base, Mn^2+^ and Mg^2+^ change the overall misincorporation rate but do not alter the relative frequencies of incoming misincorporated nucleotides. Characterization of the ion dependence of DNAP misincorporation serves as the first step towards repurposing it as a molecular recording device.

## Introduction

Traditional approaches to signal recording rely on electromagnetic radiation or electronic hardware to couple the signals of interest to an external data storage device. These approaches become cumbersome, however, when signals reside deep within complex tissues, as is the case in functional neural connectomics, where simultaneously accessing millions of neurons is currently not feasible [1]. In contrast, molecular approaches to information transfer are by nature ubiquitous, massively parallel, and inexpensive. We have recently proposed that information could be recorded onto DNA [2], [3], [4], arguably the most robust molecular information storage mechanism in nature. Recording systems based on DNA can leverage scientific and industrial interest in technologies for manipulating and sequencing nucleic acids, as well as advances in protein design.

**Figure 1:**
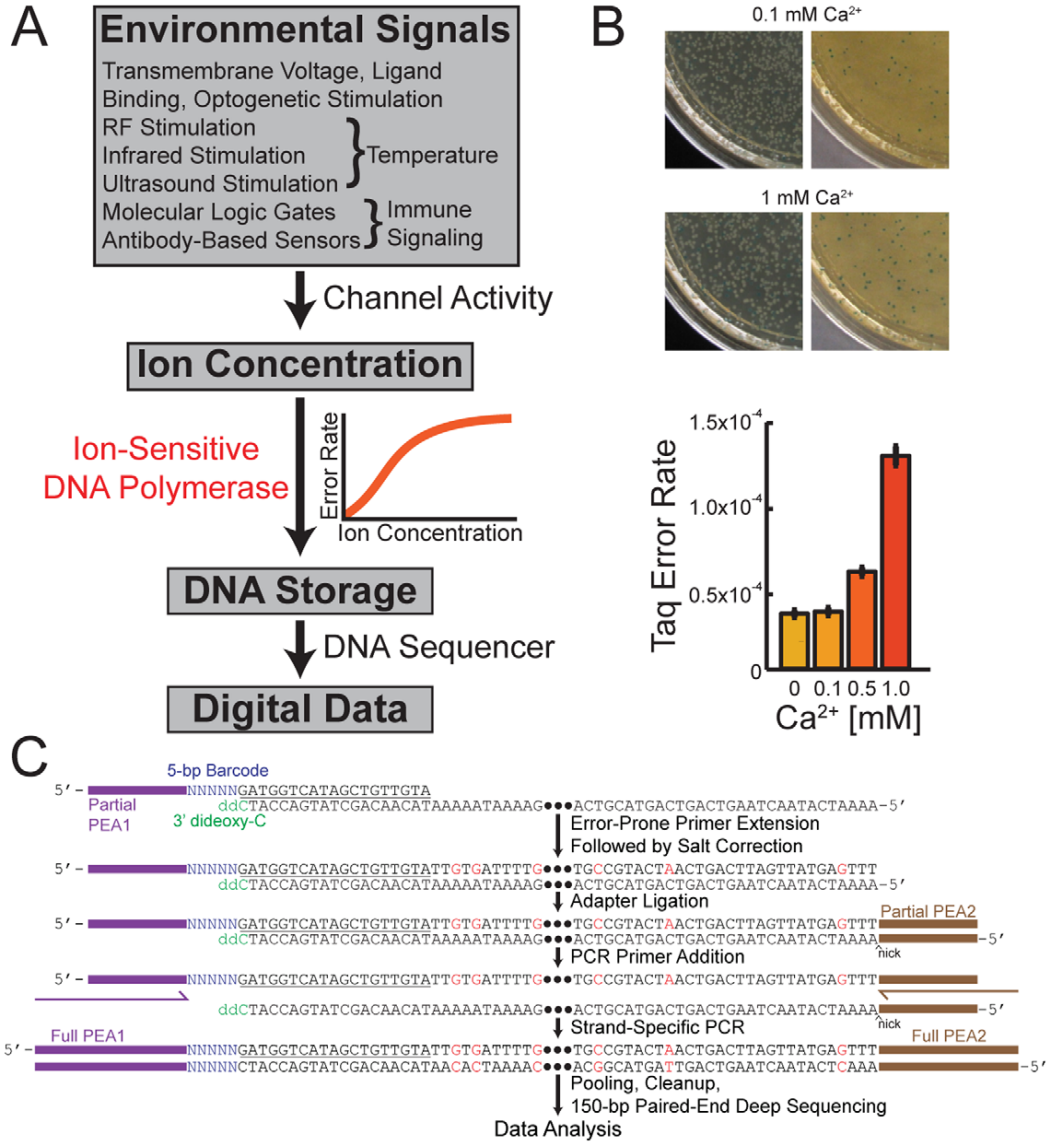
DNA polymerase (DNAP) as a molecular signal recorder. (A) Overview of a strategy for using DNA polymerases as signal recording devices. Signals (top) are coupled to intracellular or extracellular cation concentration through direct or indirect modulation of an ion channel activity. Cation concentration is in turn coupled to DNA polymerase fidelity on a known template according to a known transfer function (orange curve), generating a DNA recording, in which data is represented by the density of misincorporated bases, and which can be read by DNA sequencing (bottom). (B) Modulation of Taq polymerase by Ca^2+^ concentration, measured by a traditional blue-white colony counting assay [25]. (C) Biochemical steps of the multiplex deep sequencing assay for measuring the transfer functions of error-prone DNAPs.

A DNA polymerase could be repurposed as a nano-scale recording device, bypassing many of the hurdles of sensing technologies by locally measuring and storing information rather than requiring it to be rapidly transmitted, digitized and stored elsewhere. In a simple encoding scheme, an environmental signal of interest is coupled to the nucleotide misincorporation rate of the DNA polymerase (Figure 1A). Then, as the polymerase copies a known DNA template, the level of misincorporations produced in the copied strand will represent the amplitude of the environmental signal present. If the environmental signal varies over time, those changes could in principle also be reflected by changes in the misincorporation rate over time, enabling the DNA data storage idea to be extended to the time domain.

DNA polymerases are complex biochemical machines [5]. To establish them as molecular recording devices, it is necessary to quantify how environmental variables affect their copying fidelity. Of central importance is the *transfer function* associated with a particular DNAP, which maps the amplitude of the environmental signal to the misincorporation rate of the DNA sequence data. This transfer function is not only shaped by the biochemical properties of the polymerase, but also by other aspects of the experimental setup; it reflects the entire sensing pathway from environmental variable to filtered and processed sequence data. Therefore, the design of DNA recording devices requires the identification of any uncontrolled variables (such as local sequence context or secondary structure of the source template) that could alter the shape of this transfer function.

Cation concentrations are logical choices as the input signals for a DNA recording device because they are affected by many physiological variables, and some are known to modify DNAP fidelity [6]. Ca^2+^, for example, is involved in many signaling pathways, including neurotransmission [7] and immune activation [8], and can also be modulated by external stimuli [9]. Mg^2+^ and Mn^2+^ concentrations have been shown to strongly modulate DNAP misincorporation rate [10]. Quantifying the transfer function between cation concentration and DNAP fidelity is a useful step towards elucidating the principles of a DNA recording device.

There are a large number of known DNAPs with varying properties [11] that impact their usability as recording devices. A DNAP appropriate for DNA recording of environmental signals should ideally have a wide dynamic range of misincorporation rates and be active at mesophilic temperatures. Dpo4 (*Sulfolobus solfataricus*) [12] is a member of the Y-family of polymerases [13], [14], which are implicated in translesion bypass [15] and somatic hypermutation [16] and have high misincorporation rates. Klenow exo^−^ is the D355A, E357A mutant [17] of the Klenow Fragment of the *E. coli* DNA Polymerase I [18], which lacks 3′–5′ proofreading activity, and, unlike most commercially available DNAPs, is compatible with the 37°C extension temperature used for the Y-family enzymes. These two DNAPs seem particularly interesting in the context of recording device development.

Here we have developed a strand-specific deep sequencing method to measure the transfer function between divalent cation concentration and polymerase misincorporation rate in a highly multiplexed format. We performed barcoded, error-prone primer extensions using Dpo4 and Klenow exo^−^, at varying cation concentrations, and analyzed the products by deep sequencing. Analysis of the measured transfer functions reveals strong cation, template base, and sequence-context dependent effects on the misincorporation rate, which differ dramatically between the polymerases, and resolves the bulk misincorporation rate into its underlying transition probabilities. Our method for quantifying DNAP transfer functions will facilitate the development of engineered molecular recording devices that utilize DNA as a storage medium.

## Results

To verify that physiologically relevant ions, such as Ca^2+^, can in principle modulate DNAP fidelity, we checked the Ca^2+^ dependence of the fidelity of Taq DNAP using a *lacI^q^*-based PCR fidelity assay (Figure 1B). We constructed a derivative of pUC19 containing the *lacI^q^* repressor allele and the partial gene encoding for the colorimetric enzyme beta-galactosidase (*lacZα*). The plasmid was linearized, and PCR-amplified by Taq DNAP in buffers containing varying concentrations of Ca^2+^. Subsequently, the amplified DNA was circularized and transformed into an α-complementing strain of E.coli. Replication by Taq DNAP introduces mutations in *lacI^q^* resulting in the de-repression of *lacZα*, whose activity after complementation is detected on X-Gal indicator plates. The ratio of blue to white colonies can be used to calculate the bulk Taq error rate if the number of DNA duplications, and mutations yielding non-functional protein, are known. There are 349 single-base substitutions at 179 codons that will result in a blue phenotype in the *lacI* gene [19]. Our assay recapitulates previously reported error rates for Taq (2.6×10^−5^ bp^−1^ [20]) in the absence of added Ca^2+^, and demonstrates that increasing divalent cation concentration monotonically increased the bulk error rate.

While Ca^2+^ modulated Taq fidelity, Taq is unable to serve as a recording device, because it requires high temperatures for extension and has a low misincorporation rate (<0.015% nt^−1^) across the physiological range of Ca^2+^ concentrations [21]. We therefore focused our analysis on DNAPs that have high baseline misincorporation rates and operate at physiological temperatures.

### Multiplexed Assay for Polymerase Misincorporation

To characterize DNAPs at varying cation concentrations, we developed a multiplexed primer extension assay with deep sequencing readout (Figure 1C). Barcoded primers were first annealed to a known DNA template, followed by primer extension by the error-prone polymerase. Using a 96-well plate format allowed simultaneous testing of many cation concentrations. Subsequently, all wells were normalized to equal cation concentrations (salt correction) to eliminate ion-dependent bias in downstream biochemical steps. To eliminate bias against error-rich primer extensions, a partial Illumina adapter was then ligated downstream. Ligated products were amplified via high-fidelity PCR using primers that completed the Illumina adapter sequences. The template contained a dideoxy-C 3′ modification, preventing extension by the polymerase along the upstream primer. Consequently, the template strand did not contain the primer-binding site for PCR amplification; only strands of non-template origin were amplified, and therefore contained the full Illumina adaptors used for sequencing.

Diversity in the initial sequenced bases is required for proper cluster identification during Illumina sequencing. We therefore positioned the 5-base barcodes indexing the 96-well plate wells such that these barcodes comprised the first five bases sequenced. Following deep sequencing using the Illumina MiSeq platform, individual reads were filtered *in silico* and compared with the template sequence to quantify misincorporation rates as a function of ion concentration and base position along the template (see Materials and Methods).

This method generated hundreds to thousands of reads per cation condition, some of which were not full length (the result of abortive extensions and/or extensions containing base deletions). Duplicate plate wells with nominally equal cation concentrations and different barcode sequences were analyzed independently and used to generate misincorporation rate estimates and errors (standard error of duplicate means).

### Measurement of the Mean Transfer Function between Cation Concentration and Misincorporation Rate

**Figure 2:**
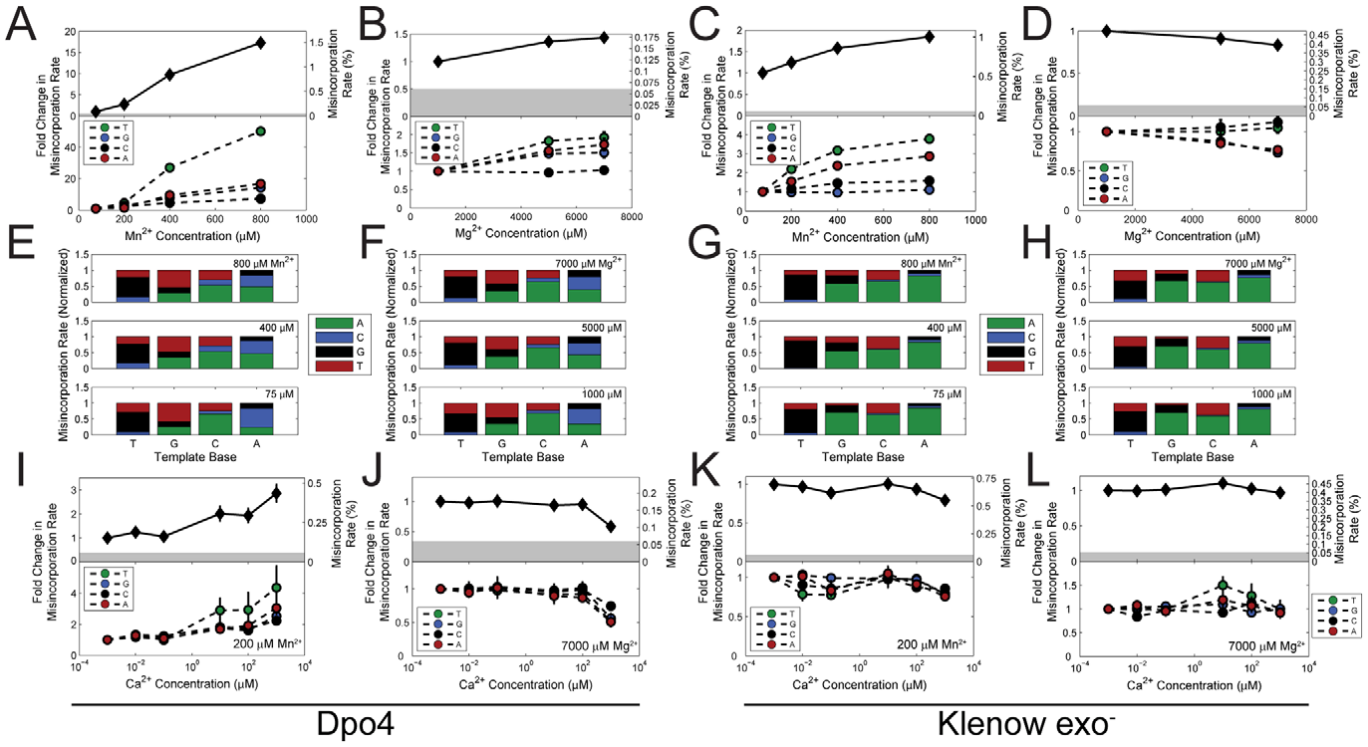
Ion-dependent misincorporation rates of Dpo4 and Klenow exo^−^ polymerases. (A, B, C, D) Mean (top) and template-base-specific (bottom) misincorporation rates as a function of Mn^2+^ (A, C) and Mg^2+^ (B, D) concentrations. (E, F, G, H) Normalized distributions of misincorporated dNTPs for each template base. (I, J, K, L) Mean (top) and template-base-specific (bottom) misincorporation rates as a function of Ca^2+^ concentration at 200 µM background Mn^2+^ (I, K) and 7000 µM background Mg^2+^ (J, L) concentrations. Errors are given in Tables S1–2, and are shown as error bars in the line graphs when they are larger than the data symbol.

We observed misincorporation rates for each reaction condition by comparing filtered sequencing reads with the known template sequence (Table S1). We first analyzed the cation dependence of Dpo4′s mean misincorporation rate, and found it to be positively correlated with both Mg^2+^ and Mn^2+^ concentrations (Figure 2A–B, top). We found that Dpo4 acts as a Mn^2+^ sensor with a gain of ∼2%/mM. Dpo4 also acts as a sensor with a gain of ∼0.01%/mM for Mg^2+^ (Table S1). Dpo4 is therefore a far better sensor for Mn^2+^ than Mg^2+^.

While the misincorporation rate for Klenow exo^−^ is also positively correlated with Mn^2+^ (top of Figure 2C), it exhibits a weak negative correlation with Mg^2+^ (top of Figure 2D). Klenow exo^−^ is a sensor for Mn^2+^ with a gain of ∼0.6%/mM and a sensor for Mg^2+^ with a gain of –0.01%/mM. Thus two cations may differ in the direction by which they modify the kinetics of misincorporation.

Note that in all cases, the measured mean misincorporation rates are much higher than the noise floor (shaded regions). This noise floor is defined as the mean plus the standard error of the mean of the misincorporation rate obtained by performing an identical protocol with the high-fidelity Phusion DNAP in HF buffer (Figure S1), and is in agreement with previous studies that measured the substitution rate of phosphoramidite synthesis [22]. Therefore, the noise floor likely results from substitution impurities in the synthetic template strands. Deep sequencing is therefore a reliable method to characterize DNAPs with high misincorporation rates.

We further measured the transfer function for mean misincorporation by Dpo4 and Klenow exo^−^ with respect to Ca^2+^ concentration. Because the kinetics of primer extension in buffers containing Ca^2+^ alone are at least ∼50 fold slower than in either Mg^2+^ or Mn^2+^ [23], we performed the primer extensions in a variety of both physiological and non-physiological Mn^2+^ and Mg^2+^ backgrounds. Misincorporation rates were only significantly affected in a small, non-physiological subset of the Mg^2+^ and Mn^2+^ backgrounds tested. The misincorporation rate by Dpo4 in a 200 µM Mn^2+^ background increases 2.9-fold from 1 nM to 1 mM Ca^2+^, the majority of which occurs between 100 nM and 1 mM (Figure 2I, Table S1). Conversely, the misincorporation rate of Dpo4 decreases by 42% between 1 nM and 1 mM Ca^2+^ in a 7 mM Mg^2+^ background, with virtually all of the change occurring between 100 µM and 1 mM Ca^2+^ (Figure 2J). Ca^2+^ has no effect on misincorporation rate with Klenow exo^−^ in the same backgrounds (Figure 2K and 2L) nor in most other enzyme/buffer combinations (Table S1). Therefore neither of the tested DNAPs is promising as a Ca^2+^ sensor without further modifications.

### Base Specificity of Misincorporation

The misincorporation characteristics of DNAPs depend not only on cation concentrations, but also on the particular template base being copied. Deep sequencing allows quantification of the misincorporation rate at every position within the template (Figure 2A–D). Note that misincorporation by Dpo4 opposite a template T exhibits a >50-fold increase over the range of Mn^2+^ studied, while misincorporation rates opposite other bases show a comparatively weak dependence on Mn^2+^ (Figure 2B, Table S1). Thus the mean Mn^2+^ dependence of misincorporation rate of Dpo4 is largely driven by misincorporations opposite T. There is no obvious correlation of the misincorporation rate with the identity of the base preceding the template base (Figure S2).

Deep sequencing also allows direct measurement of the 4×4 transition probability matrix between template base and incorporated base (Table S2, Figure 2E–H). For example, the disproportionate Mn^2+^ dependence of misincorporation by Dpo4 opposite template T is largely due to misincorporation of dGTP. Likewise, mutations caused by Klenow exo^−^ are generally dominated by misincorporation of dATP, except on template T, which shows a >4-fold preference for misincorporation of dGTP. Misincorporation by Dpo4 of dGTP opposite template T increases 50-fold with Mn^2+^. Note, however, that the relative proportions of the misincorporated bases on a given template base are largely insensitive to cation concentration for both Dpo4 and Klenow exo^−^. Rather, cation concentration acts as a scaling factor with respect to misincorporation opposite a given template base; it is the differential magnitude of this scaling factor between the template bases that underlies the template base dependence of misincorporation.

### Misincorporation is Context-Dependent

**Figure 3:**
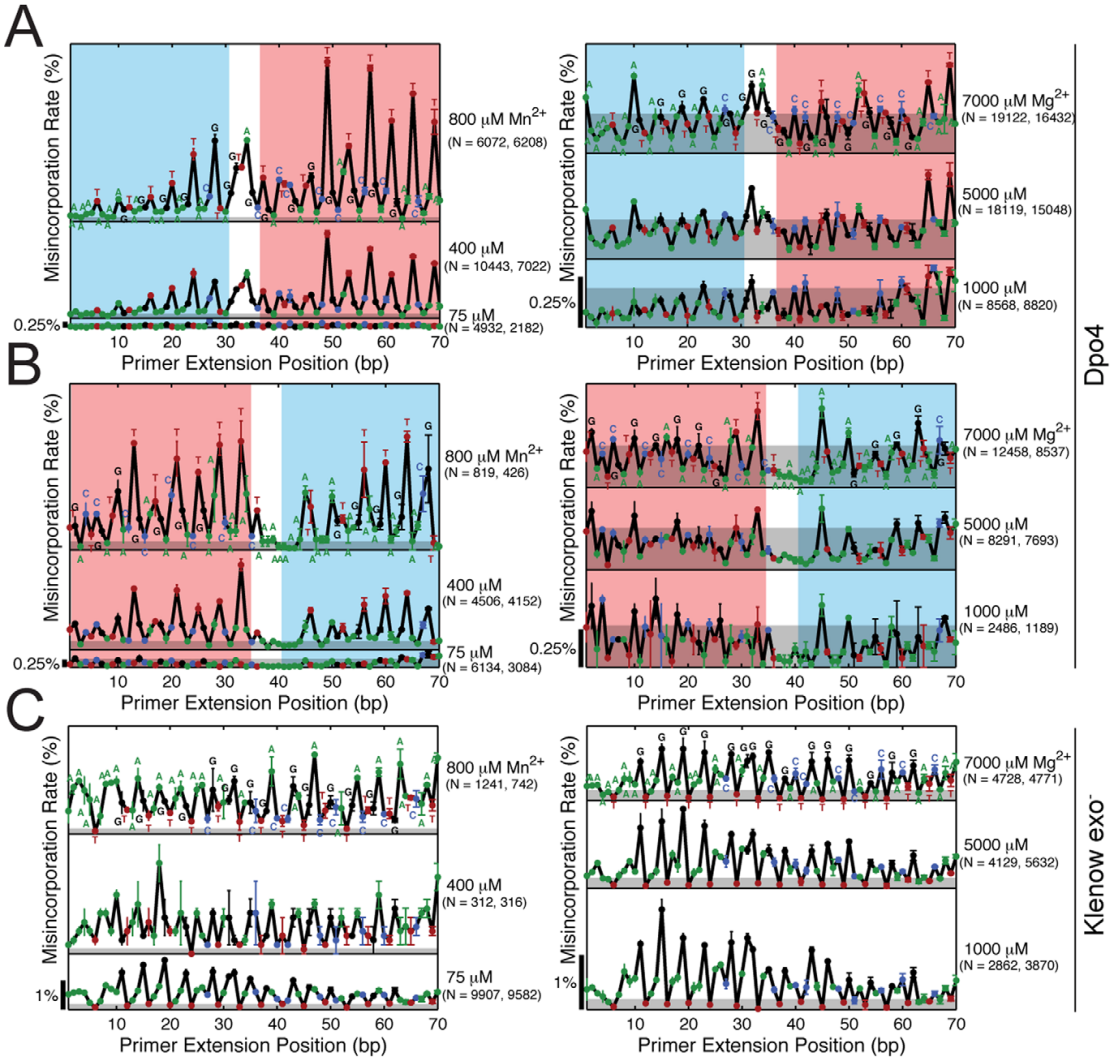
Template position dependence of misincorporation rates. (A) Template position dependence of Dpo4 misincorporation rates on the original template at varying Mn^2+^ (left) and Mg^2+^ concentration (right). (B) Template position dependence of Dpo4 misincorporation rates on the swapped template at varying Mn^2+^ (left) and Mg^2+^ concentration (right). (C) Template position dependence of Klenow exo^−^ misincorporation rates on the original template at varying Mn^2+^ (left) and Mg^2+^ concentration (right). Letters above each data point denote the identity of the template base at that position. Grey shaded areas indicate the noise floor, defined as the maximum over positions of the misincorporation rate (plus SEM) observed in an identical experiment with Pfusion HF DNA polymerase (Figure S1). Red (blue) shaded areas in (A) and (B) correspond to shared sub-sequences between the original and the swapped template.

Cations change misincorporation probabilities but not the distribution of misincorporations across incoming dNTPs. However, the template base itself is not, in general, sufficient to predict misincorporation rate; the sequence context is important as well (Figure 3A–C). For Dpo4, the shape of the graph is dominated by preferential misincorporations at template T (red dots). The dependency on the sequence, however, is complicated: switching the first half of the template (shaded blue) with the second half (shaded red) leaves some aspects of the misincorporation curve similar while changing others. Indeed, the swapped template leads to a more even distribution of misincorporations, indicating that template choice is an important design parameter for DNA recording.

There is no obvious sequence context dependence of misincorporation for Klenow exo^−^ (Figure 3C), beyond the identity of the template base. Curiously, the misincorporation rate opposite template G, which dominates at 75 µM Mn^2+^, stays relatively unchanged with increasing Mn^2+^ concentration, while misincorporations opposite template A increase, becoming the predominant peaks at 800 µM Mn^2+^. Thus different DNAPs are differently affected by both cation concentrations and local sequence contexts.

### Statistical Analysis of Misincorporation Events

Our deep sequencing method produces large datasets that can be used to characterize the correlations within each strand of synthesized DNA, as well as the statistical distributions across strands. To test the hypothesis that DNAPs could tend to string errors together, we analyzed the lag-one correlations of misincorporations, asking if a misincorporation on one base makes it more likely that there is a misincorporation on the next base. After correction for bias due to correlations within the template itself (see Materials and Methods), there is a weak but statistically significant correlation of misincorporations across bases for Klenow exo^−^ at 800 µM Mn^2+^ (0.047±0.002% excess misincorporations per base). For Dpo4, misincorporations at consecutive positions appear independent from one another (all excess errors <0.01% per base). Therefore, only for Klenow exo^−^ is a misincorporation on a base associated with an increased probability of misincorporation on the next base.

It is unknown to what extent molecular heterogeneity plays a role in the generation of DNAP misincorporations. If each DNAP molecule performs misincorporations according to the same statistics, the distribution of the total number of misincorporations per read should be governed by a Poisson distribution. The variance is larger than the mean, however, for each DNAP/template combination tested, and the null-hypothesis of a Poisson distribution can be rejected for each of the datasets (*Χ* ^2^ test, *p*<0.05). Thus the ensemble of nominally identical DNAP molecules is heterogeneous with respect to misincorporation rate.

**Figure 4:**
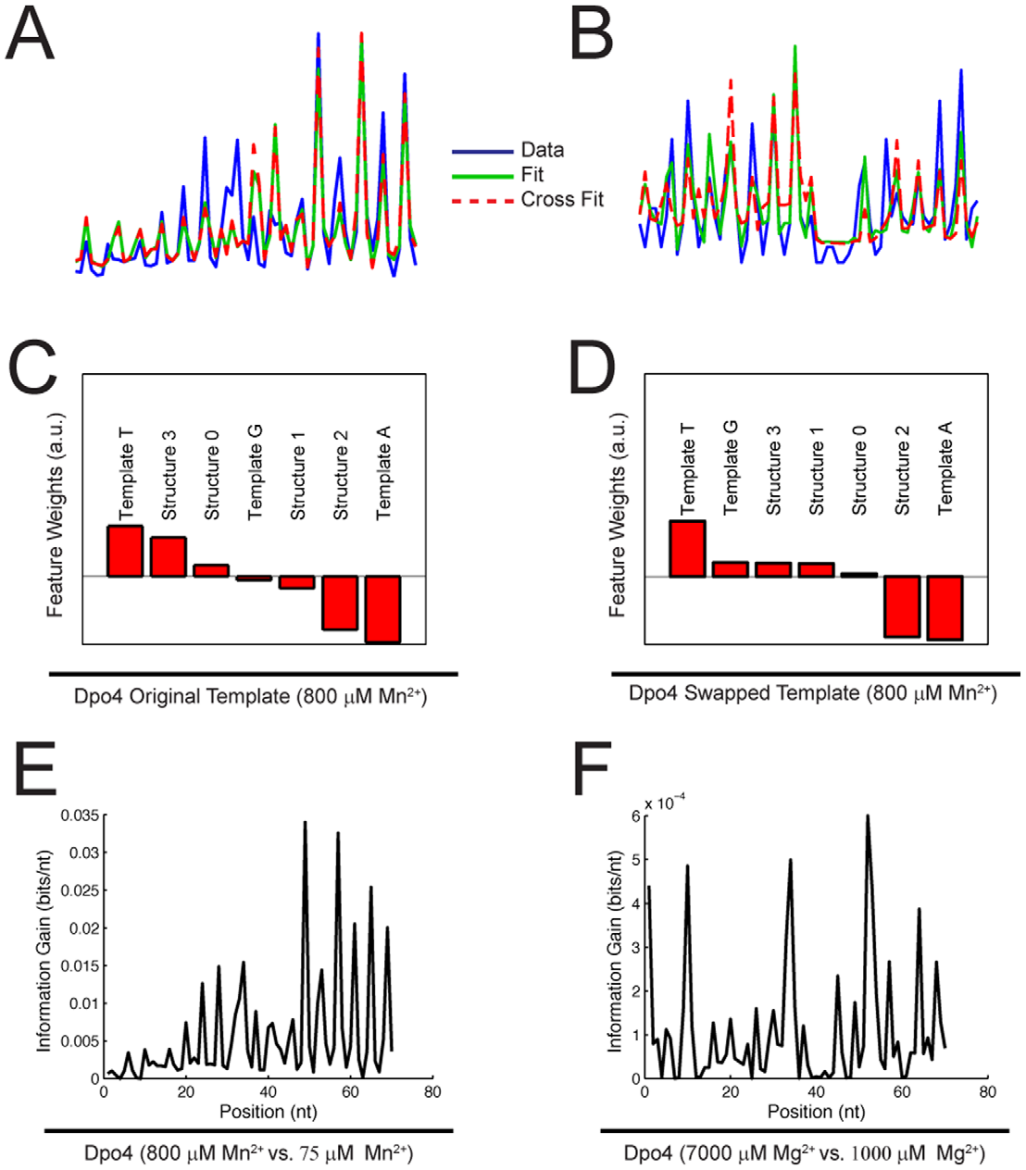
Statistical analysis of misincorporation by Dpo4. (A) Spatial dependence (un-normalized) of Dpo4 error rate at 800 µM Mn^2+^ on the original template (blue curve), and generalized linear model fits of this data set with respect to itself (green curve), and with respect to the swapped template data set (red curve). (B) Spatial dependence (un-normalized) of Dpo4 error rate at 800 µM Mn^2+^ on the swapped template (blue curve), and generalized linear model fits of this data set with respect to itself (green curve), and with respect to the original template data set (red curve). (C) Feature weights for generalized linear model fit to Dpo4 original template data. (D) Feature weights for generalized linear model fit to Dpo4 swapped template data. (E) Information gain per base as a function of template position, for discrimination between high (800 µM) and low (75 µM) Mn^2+^ by Dpo4. (F) Information gain per base as a function of template position, for discrimination between high (7000 µM) and low (1000 µM) Mg^2+^ by Dpo4.

To further study the determinants of misincorporation, we fit the misincorporation data set to a generalized linear model (GLM) containing sequence features that could plausibly impact misincorporation rates (Figure 4). Possible features included the identity of the template base and the predicted regional secondary structure. The models were able to fit the data (*R* ^2^ = 0.58±0.02 and 0.53±0.11 for the original and swapped templates, respectively, Figure 4A and 4B). Interestingly, the models captured the interplay of sequence properties that determine the spatial dependence of misincorporation. Fits to the original template data could predict the spatial dependence of misincorporation on the swapped template (*R* ^2^ = 0.49±0.06), and vice versa (*R* ^2^ = 0.50±0.01). Furthermore, the weights assigned to different features (Figure 4C and 4D) in the model point to potential determinants of the error rate. For example, the models identify the positive contribution of a template T to Dpo4′s error rate and also suggest that local secondary structure may play a role.

### Information Content of Misincorporations

Because cation concentration modulates the number of misincorporations in the copied DNA, one can consider the sequenced data to store information about the cation concentration present during primer extension [24]. The information gain per base is related to the likelihood that the observed misincorporation rate at a given template position was produced at a particular cation concentration. For Dpo4 at high (800 µM) vs. low (75 µM) Mn^2^+, the most informative template bases transmit ∼0.03 bits of information per base about Mn^2+^ concentration (Figure 4E), whereas only ∼5×10^−4^ bits per base are transmitted at high (7000 µM) vs. low (1000 µM) Mg^2+^. Therefore, in the limit in which Mn^2+^ concentration could be modulated as each nucleotide is added, a Dpo4-based DNA recording device could in principle write 11 megabytes onto a template the length of a human genome (3.2×10^9^ bases).

## Discussion

In this work, we have developed a method that can quantitatively map the misincorporation landscapes of error-prone polymerases as a function of environmental signals. Specifically, we quantified how the concentrations of environmental Mg^2+^, Mn^2+^ and Ca^2+^ affect the fidelity of Dpo4 and Klenow exo^−^. Mn^2+^ has the strongest influence on misincorporation rates in comparison to the other cations. Our method resolves the misincorporation by spatial position and nucleotide-to-nucleotide transition. We find that, for Dpo4 and Klenow exo^−^, Mn^2+^ and Mg^2+^ change misincorporation rates but leave the distribution across incoming misincorporated nucleotides untouched. We have further shown that polymerase misincorporation rates exhibit sequence dependences. The development of a DNAP-based cation sensor, then, necessitates calibration of misincorporation rates at specific template positions, within specific sequence contexts, and at specific buffer conditions. The buffer-specificity of some DNAPs suggests that polymerase-based sensors might work best within controlled buffer environments, e.g. within living cells expressing ion channels, which can maintain buffer integrity, but selectively allow targeted ions to permeate. Our experiments quantify the transfer function of misincorporation from cations, through processing, all the way to DNA sequence data.

Our assay differs in important ways from the bacterial assays that have been used for the quantification of DNAP behavior [25]–[27]. Through deep sequencing we can readily observe polymerase trajectories with single molecule and single base resolution while simultaneously generating large datasets, both of which are critical for achieving the comprehensive analyses necessary for establishing polymerase data encoding transfer functions. Single base pair resolution allows quantifying the template dependence of misincorporations, while single molecule resolution allows quantification of the correlation structure of misincorporations.

The method introduced here does have limitations, some of which can be mitigated. For example, the measured background noise level is likely dominated by errors introduced during the chemical synthesis of the oligonucleotides used as templates. The use of clonal isolates should dramatically lower that noise level and may prove necessary in adapting this method to the characterization of high fidelity DNAPs. In addition, GLM analysis indicates that the spatial dependence of the observed misincorporation rates may be in part due to the secondary structure of the ssDNA template. Using a nicked, double stranded template would reduce this source of variance, but would limit the applicability of the method to DNAPs with strand displacement or nick translation activity. While sophisticated molecular counting methods [28] and clonal substrates are necessary to quantify the low misincorporation rates of proofreading polymerases using sequencing [22], in this study, we have investigated error-prone polymerases, and are therefore readily able to measure strong effects despite the limitations of our method.

Certain limitations of the method cannot be mitigated without resorting to engineered polymerase variants. For example, we have shown that neither DNAP studied here can act as a Ca^2+^ sensor in physiologically relevant conditions. Furthermore, these biologically-based recording devices are limited to conditions that enable efficient enzymatic activity; such devices will not work, without modification, in environments of extreme pH, temperature, oxidative stress, proteolysis, etc.

While we have demonstrated how a static ion concentration can be measured by a polymerase copying DNA, it would ultimately be useful to have polymerase-based sensors for time-dependent as well as static signals. To do so, it will be necessary to optimize the sensing polymerase for speed (for temporal resolution), processivity (for recording time), low pause probability (for linearity of temporal readout), total misincorporation rate (for information density) and dynamic range of misincorporation rate (for signal to noise ratio). We have shown that divalent ion concentration can be a potent, yet continuously tunable, modulator of polymerase misincorporation rates, and that such modulation can be restricted to particular template bases and base-to-base transitions. Based on its >15-fold change in misincorporation rate over the Mn^2+^ range tested here, Dpo4 could act as a high resolution Mn^2+^ sensor. The fact that misincorporations are largely localized to certain template bases makes it possible in principle to preserve relevant features of the template (on the non-error-prone template bases) while transmitting information at the same time (on the other bases).

Advances in fields such as neuroscience impose spatial, temporal, and combinatorial challenges of unparalleled scope, associated with the three-dimensional recording and analysis of complex cellular systems. A molecular device capable of measuring and recording sub-cellular signals, which can be manufactured and delivered to target environments in a scalable fashion, may emerge as an optimal platform for biological signal recording. However, the basic principles for designing and testing such molecular recording devices *in vitro* have not yet been established. This study measures a static environmental signal – divalent cation concentration – by using DNA polymerases as molecular recording devices. The synthesized DNA strand can be considered as an archival medium, which stores the measured signal in the form of a misincorporation rate with respect to the known template. Indeed, the use of DNA as an information storage medium leverages the rapid improvement of sequencing technology, which is currently outpacing the Moore's law rate of improvement of microelectronic technology [29], and which promises to make DNA sequencing a preferred method for extracting data from biological and bio-molecular systems [30], [31]. Extension of the techniques described here to time-varying signals and engineered polymerases could lead to molecular sensing technologies of unprecedented scalability.

## Materials and Methods

### Reagents

All primers were synthesized by IDT. All enzymes, dNTPs and buffers were from New England Biolabs (NEB) unless otherwise indicated.

### Measurement of the Misincorporation Rate of Taq Polymerase

A derivative of pUC19 containing the *lacZα* and *lacI^q^* allele was linearized with DraII. Linearized DNA was purified and used as template in PCR reactions containing 5 U Taq DNAP, standard Taq buffer with 1.5 mM Mg^2+^, 200 µM dNTPs (Invitrogen), CaCl_2_ to indicated concentrations and 0.5 µM each of the primers CLA55 (5′-AGCTTATCGATAAGCGATGCCGGGAGCAGACAAGC-3′) and CLA33 (5′-AGCTTATCGATGGCACTTTTCGGGGAAATGTGCG-3′). Reactions were cycled 30 times with 1 minute of annealing at 55°C and 4.5 minutes extension at 68°C. PCR products were purified using a DNA Clean and Concentrator-5 kit (Zymo Research). After determining the A_260_, the amplified DNA was digested at 37°C for 4 h with 10 U ClaI, and purified. Ligation were performed using the NEB quick ligation kit with 50 ng of DNA, and directly transformed into DH5α *E. coli* and plated on LB-Carb containing 40 µg/μL X-Gal. Blue and white colonies were counted after incubation at 37°C overnight.

The error rate *f* was calculated as *f*  =  -ln(*F*)/(*db*) [32], where *F* is the fraction of white colonies, *d* is the number of DNA duplications and *b* = 349 bp is the effective target size of the 1080 bp *lacI* gene [19]. Error bars for the blue-white screening experiment were obtained using Poisson statistics where, for large *n*, the distribution is approximately Gaussian with a variance that is identical to the mean.

### Primer Extension Assay

All reactions were performed in 96-well plates, on ice, unless otherwise noted. Annealing reactions were performed by mixing 100 nM barcoded primer N1.1.1.x (ACACTCTTTCCCTACACGACGCTCTTCCGATCTNNNNNGATGGTCATAGCTGTTGTA, where the underlined region is the unique 5-mer barcode for each reaction, and x = 1 to 96; barcodes were composed with pairwise Levenshtein distances greater than one) with 150 nM PAGE-purified original template strand N1.0.6 (5′-AAAATCATAACTAAGTCAGTCAGTACGTCAGTAGCTCAGTCGATGGATGCAATGAATGAATGAATGAAAATAAAAA TACAACAGCTATGACCAT-ddC-3′) or swapped template strand N1.0.8 (5′-CGATGGATGCAATGAATGAATGAATGAAAATAAAAAAAAATCATAACTAAGTCAGTCAGTACGTCAGTAGCTCAGTTACAACAGCTATGACCAT-ddC-3′) in 1× annealing buffer (Table S3). The primer and template oligonucleotides were annealed by incubation at 95°C for 5 min, followed by a −0.1°C/sec ramp until reaching 25°C. The PEA2 adapter dsDNA was made at the same time, by mixing N1.2.1 (5′-P-AGATCGGAAGAGCGGTTCAGCAGGAATGCCGAG-3′) and N1.2.2 (5′-CTCGGCATTCCTGCTGAACCGCTCTTCCGATCT-3′) to a final concentration of 300 nM each and annealing them via the same protocol.

Primer extensions were performed as per the manufacturer's instructions (Dpo4, Klenow exo^−^, Phusion) in 10 µL reactions containing 1 µL annealing reaction, 50 µM each dNTP, and 1 µL of a 1∶1000 dilution of Dpo4 (Trevigen) in Dpo4 annealing buffer, 1 µL Klenow exo^−^, or 5 µL 2× Phusion Mastermix in HF buffer, in 1× extension buffer (Table S3). Primer extensions were initiated with the addition of divalent cation (chloride salt) to the reaction mixture and incubation at 37°C for 1 h, except for Phusion, which was incubated at 95°C for 10 minutes followed by 72°C for 1 h.

After primer extension, a 10 µL mixture of divalent cations was added to each well such that the final concentration in each well was normalized to 800 µM Mn^2+^, 7 mM Mg^2+^ and 1 mM Ca^2+^. An automated liquid handling robot (Agilent) was used to create stocks of the divalent cations used for primer extension and salt correction in a 96-well plate format.

Ligations were performed in 10 µL volumes containing 6 µL salt-corrected primer extensions, 200 U/μL T4 DNA ligase (New England Biolabs), 1 mM ATP, and 1.23 nM PEA2 adapter. Ligations reactions were incubated at 16°C overnight.

High-fidelity PCR of the ligation reactions was performed by adding 5 µL ligation reaction, 0.5 µM primer N1.3.1 (5′-CAAGCAGAAGACGGCATACGAGATCGGTCTCGGCATTCCTGCTGAACCGCTCTTCCGATCT-3′) and 0.5 µM N1.3.2 (5′-AATGATACGGCGACCACCGAGATCTACACTCTTTCCCTACACGACGCTCTTCCGATCT-3′), in 1× HF Phusion mastermix, and incubating at 98°C for 30 s, followed by 30 cycles of incubation at 98°C for 10 s and 72°C for 1 m, followed by a final extension at 72°C for 10 m.

### DNA Sequencing

Pooled PCR products were cleaned using a MinElute Cleanup Column (Qiagen) into 20 µL buffer EB, resulting in a final concentration of 300–400 ng/uL. Cleaned products were diluted to a nominal concentration of 12–14 nM, calculated using a droplet spectrophotometer (Qubit, Invitrogen), assuming a nominal average dsDNA length of 100 bp in the sample. The diluted sample (2 µL) was combined with 8 µL water, denatured with 10 µL NaOH and added to 980 µL HT1 buffer (Illumina). To introduce sufficient base diversity for baseline intensity correction during the sequencing run, 600 µL phiX paired-end library DNA (Illumina) was combined with 400 µL of the sample and loaded on a MiSeq (Illumina) for 150 bp paired-end sequencing. Approximately 4–5 pm of sample and at least 5 pm of phiX DNA were loaded in each sequencing run.

### Data Analysis

Raw sequencing reads in the forward direction were filtered for the presence of the left primer binding sequence, the first 12 bp of the right adaptor sequence, and the presence of a correct barcode. Raw sequencing reads in the reverse direction were filtered for the presence of the left primer binding sequence and the barcode. Forward reads in which the sequence between the left and right adaptors did not exactly match the corresponding reverse paired end read were discarded. We also filtered out instances of a short spurious PCR product resulting from known primer dimer contamination. The raw sequence reads are available for download (NCBI accession number SRP014521). The forward reads thus filtered were aligned with the sequence of the theoretical error-free primer extension product (reverse complement of the template) using the BioPython function pairwise2. align. globalxs with gap open and gap extend penalties of −10 and −2 respectively. Sequences with length greater than or equal to 70 bases between the left and right adaptors, and alignment scores greater than 60, were selected for further analysis. Misincorporations aligned to a given template position were counted towards the tally of misincorporations at that position and with respect to its corresponding template base. Misincorporation rates were measured as ratios of the number of misincorporations at a given position or template base to the total number of events counted at that position or template base. Insertions or deletions at a given position were not counted towards the misincorporation tally nor towards the tally of total events at a position. An alternative analysis method that did not rely on alignments was also used (**Text S1** and **Figure S3**). All data analysis was performed in Python and Matlab; code is available upon request.

### Generalized Linear Model (GLM) Construction, Poisson Statistics and Auto-Correlations

Generalized linear models (GLMs) were constructed to predict the misincorporation probability at a given template position based on sequence context and secondary structure. To construct the variable to be fit (y), we took the filtered, aligned reads and removed those that contained insertions or deletions, resulting in a set of 70 nt long alignments to the first 70 bases of the template. We further ignored the first and last 3 bases of these alignments to enable the use of regional information on secondary structure. For each base in y, the regressor contained binary features representing the identity of the template base, a continuous feature representing the position in the template, and the regional secondary structure prediction at positions ranging from three bases before the template base to three bases after. Only three of the four template bases were used as explicit features, as the fourth is included in the bias term. The ensemble-averaged secondary structure of the original and swapped templates were calculated at 37°C and standard salt conditions using NuPack software [33]. The secondary structure at a given template position was defined to be the sum of the ensemble pair probabilities of the corresponding template base with respect to all other template bases, and was calculated as one minus the probability that the corresponding template base is unpaired, as evaluated by NuPack. The data sets used for GLM fitting corresponded to individual experimental replicates. GLM calculations were performed using the Matlab glmfit function with a binomial distribution. Excess lag-one errors were calculated by subtracting the error expected based on the misincorporation probability *(n_p_/N_t_)^2^*, where *n_p_* is the number of errors at a particular template position within the data set, and *N_t_* is the total number of templates in the data set.

### Calculation of the Shannon information Gain Per Base

Calculation of the information gain per base proceeded by a Bayesian framework. Initially equal prior probabilities were assigned to high and low cation concentrations, corresponding to one bit of missing information, i.e., *p(L)*  =  *p(H)*  = ½, where *p(L)* and *p(H)* are the probabilities that the cation concentration is in the low state or high state, respectively. Observing the misincorporation rate updated the distribution. The expected information gain (conditional entropy) is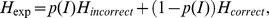 where *p(I)* is the probability of misincorporation weighted by the prior over cation concentration (see below), and *H_incorrect_* and *H_correct_* are conditional Shannon entropies, defined as



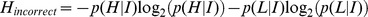



and
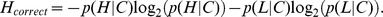



By Bayes' rule, *p(H|I)  =  p(I|H)p(H)/p(I)*, where *p(I|H)* is the misincorporation rate per base at high cation concentration, as shown in Figure 3. The other conditional probabilities (*p(H|C), p(L|I), and p(L|C)*) were calculated analogously. The misincorporation probability was then calculated through marginalization, e.g., *p(I)  =  p(I|H)p(H) + p(I|L)p(L)*. Inserting these expressions into the equation for expected information gain (*H_exp_*) allowed for calculation of the number of bits of information gained per base.

## Supporting Information

Figure S1 **Measurement of the experimental noise floor.** The spatial distribution (top) and template-base-specific (bottom) misincorporation rates for Phusion on the original (A) and swapped (B) templates. (C) Misincorporation rates for Phusion on the original template, using a modified protocol in which the ligation products were pooled and cleaned before high-fidelity PCR amplification. Dashed lines indicated the maximum peak, plus the error, of the spatially-distributed misincorporations (top) or the mean + SEM of misincorporations across all template bases (bottom) misincorporations, and served as the noise floors in the main text.
(TIF)

Figure S2 **Analysis of misincorporation at two-base motifs in the template sequence.** Misincorporation rate as a function of the template base and of the base preceding the template base, for Dpo4 at 800 µM Mn^2+^ on the original (A) and swapped (B) templates.
(TIF)

Figure S3 **Comparison of sliding window and alignment-based analyses.** Comparison of alignment-based (main text) and sliding window-based (Text S1) analyses of the spatial distribution of Dpo4 (A) and Klenow exo^−^ (B) misincorporation rates at varying Mn^2+^ (left) and Mg^2+^ (right) concentrations.
(TIF)

Text S1 **Alternate Misincorporation Analysis.** An analysis that uses a sliding window, as opposed to sequence alignments (main text) to determine misincorporation rates.
(DOC)

Table S1 **Mean Misincorporation Rates.** The misincorporation rate and fold-change in misincorporation rate for all conditions studied. Mean misincorporation rates are the average over the four template bases. The fold-change misincorporation rate is with respect to the lowest cation concentration in the titration.
(XLS)

Table S2 **Base-Transition Probabilities.** The incorporation rate of each dNTP opposite each template base, for all conditions studied.
(XLS)

Table S3 **Primer Extension Assay Buffers.** Buffers used for annealing and extension for each of the DNAPs studied.
(XLS)
