# Supplementary figures and images for: Measuring Cation Dependent DNA Polymerase Fidelity Landscapes by Deep Sequencing
Source: PLoS One. 2012 Aug 22;7(8):e43876. doi: 10.1371/journal.pone.0043876 (PMC3425509; doi:10.1371/journal.pone.0043876)

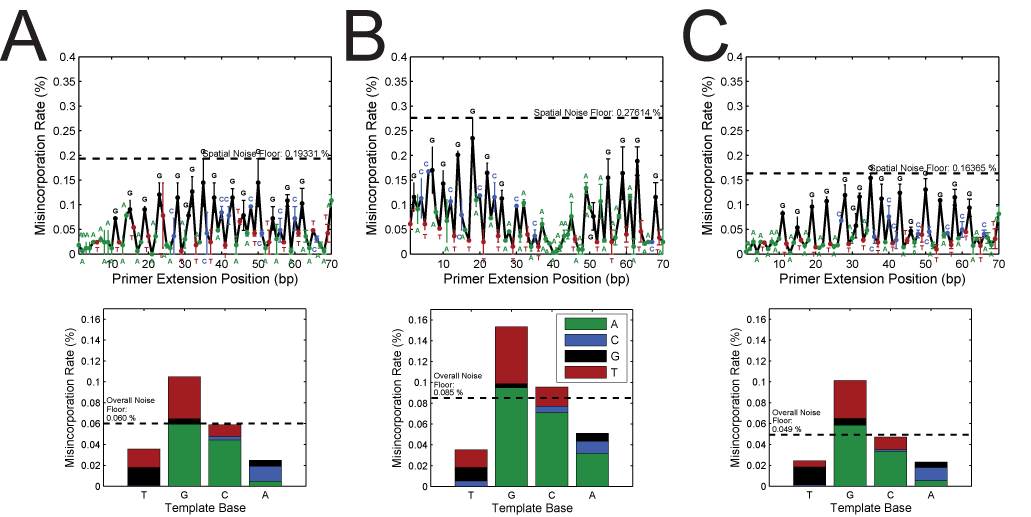

Supplement: Figure S1 — Measurement of the experimental noise floor. The spatial distribution (top) and template-base-specific (bottom) misincorporation rates for Phusion on the original (A) and swapped (B) templates. (C) Misincorporation rates for Phusion on the original template, using a modified protocol in which the ligation products were pooled and cleaned before high-fidelity PCR amplification. Dashed lines indicated the maximum peak, plus the error, of the spatially-distributed misincorporations (top) or the mean + SEM of misincorporations across all template bases (bottom) misincorporations, and served as the noise floors in the main text. (TIF) [file pone.0043876.s001.tif]

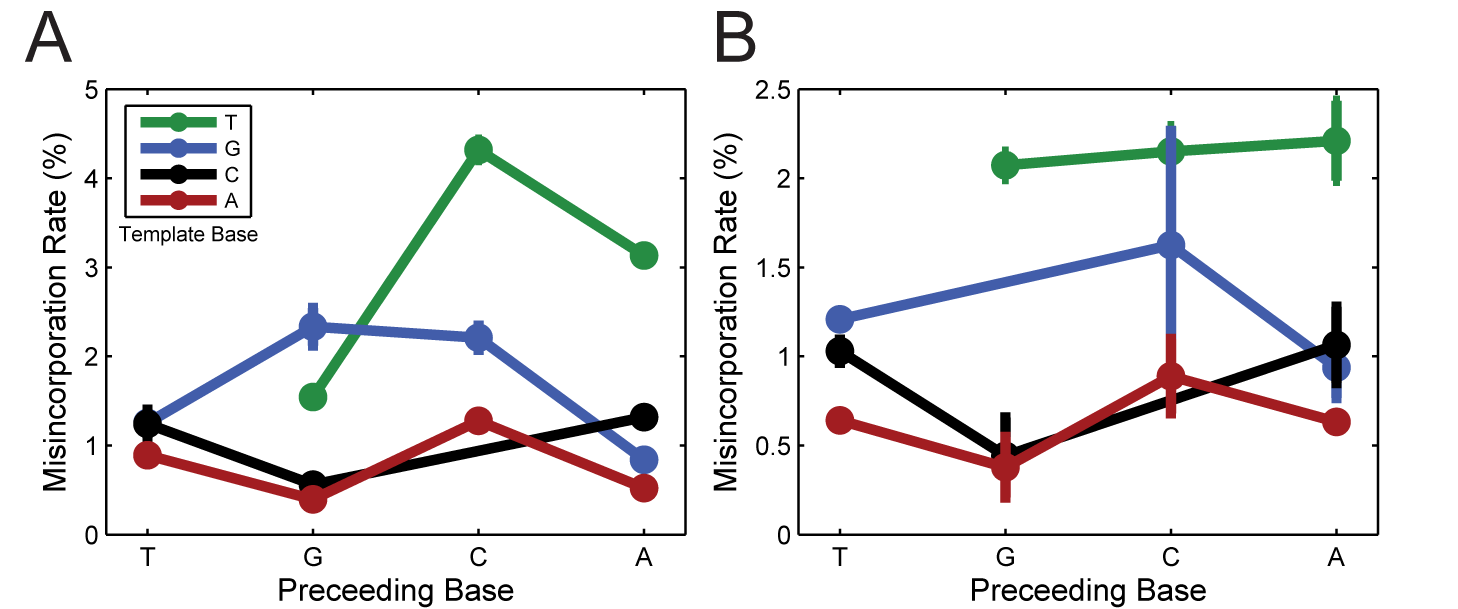

Supplement: Figure S2 — Analysis of misincorporation at two-base motifs in the template sequence. Misincorporation rate as a function of the template base and of the base preceding the template base, for Dpo4 at 800 µM Mn2+ on the original (A) and swapped (B) templates. (TIF) [file pone.0043876.s002.tif]

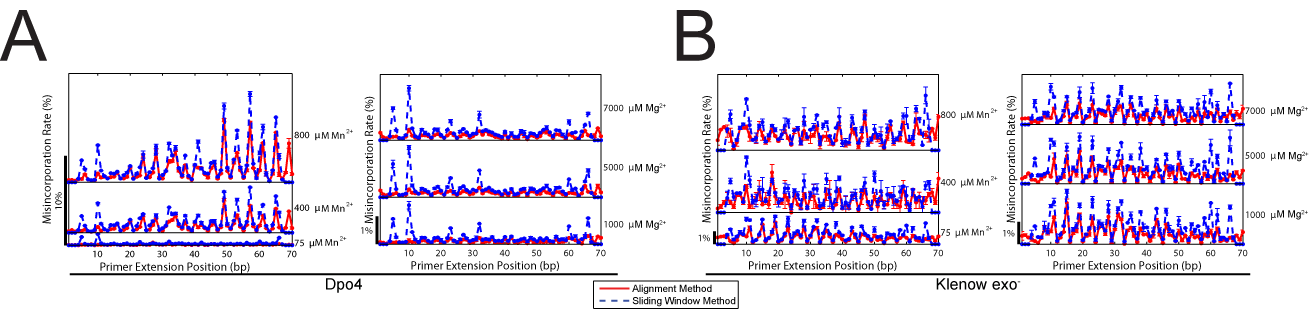

Supplement: Figure S3 — Comparison of sliding window and alignment-based analyses. Comparison of alignment-based (main text) and sliding window-based (Text S1) analyses of the spatial distribution of Dpo4 (A) and Klenow exo− (B) misincorporation rates at varying Mn2+ (left) and Mg2+ (right) concentrations. (TIF) [file pone.0043876.s003.tif]
