# Supplementary material for: Measuring Cation Dependent DNA Polymerase Fidelity Landscapes by Deep Sequencing
Source: PLoS One. 2012 Aug 22;7(8):e43876. doi: 10.1371/journal.pone.0043876 (PMC3425509; doi:10.1371/journal.pone.0043876)
Supplement: Text S1 — Alternate Misincorporation Analysis. An analysis that uses a sliding window, as opposed to sequence alignments (main text) to determine misincorporation rates. (DOC) [file pone.0043876.s004.doc]

Measuring Cation Dependent DNA Polymerase Fidelity Landscapes by Deep Sequencing

# Text S1

Brad Zamft*, Adam Marblestone*, Konrad Kording, Daniel Schmidt, Daniel Martin-Alarcon, Keith Tyo, Ed Boyden and George Church

### Alternate Misincorporation Analysis (Sliding Window)

The analysis described in the main text relies on sequence alignments to compare sequence reads with the known template. Alignments, however, require the sequenced read to be of sufficient length that the alignment algorithm can work reliably with respect to the full-length templates. We therefore imposed a length cutoff of 70 bp, as well as an alignment score cuttoff, to ensure that the sequenced reads could be properly aligned. As a control for analysis methodology, we also developed an alternate analysis method which does not make use of sequence alignments and which applies to both short and long sequences. Here, the filtered forward reads were compared with the perfect product sequence using a sliding window based on absolute position indexing. We counted a particular absolute base position in a particular read if the three bases before it and the three bases after it matched their respective template sequences. For such valid positions, misincorporation values were tallied with respect to the template. This method is not applicable to the first three bases and last three bases of the primer extension, and therefore the misincorporation rates at these six positions were set to zero for clarity in plotting. The results from such analyses on Dpo4 and Klenow exo^-^ were consistent with the analyses given in the main text (Figure S3). This method, however, effectively removes most sequences with insertions or deletions from the analysis, and creates bias against sequences with multiple misincorporations within the sliding window.

All figures in the main text used the alignment based method, which has also been used in previous studies [32].
